# Monitoring of HIV treatment in seven countries in the WHO Region of the Americas

**DOI:** 10.2471/BLT.14.147447

**Published:** 2015-05-27

**Authors:** Pablo F Belaunzarán-Zamudio, Yanink N Caro-Vega, Bryan E Shepherd, Brenda E Crabtree-Ramírez, Paula M Luz, Beatriz Grinsztejn, Carina Cesar, Pedro Cahn, Claudia Cortés, Marcelo Wolff, Jean W Pape, Denis Padgett, Eduardo Gotuzzo, Catherine McGowan, Juan G Sierra-Madero, on behalf of CCASAnet

**Affiliations:** a Clínica de Inmuno-Infectología, Departamento de Infectología, Instituto Nacional de Ciencias Médicas y Nutrición Salvador Zubirán, Calle Vasco de Quiroga 15, Colonia Belisario Domínguez Sección XVI, Delegación Tlalpan, México Distrito Federal, CP 14080, Mexico.; b Department of Biostatistics, Vanderbilt University, Nashville, United States of America (USA).; c Instituto de Pesquisa Clínica Evandro Chagas, Fundacão Oswaldo Cruz, Rio de Janeiro, Brazil.; d Fundación Huésped, Buenos Aires, Argentina.; e Fundación Arriarán, Universidad de Chile, Santiago, Chile.; f Le Groupe Haitien d'Etude du Sarcome de Kaposi et des Infections Opportunistes, Port-au-Prince, Haiti.; g Instituto Hondureño de Seguridad Social and Hospital Escuela, Tegucigalpa, Honduras.; h Instituto de Medicina Tropical Alexander von Humboldt, Lima, Peru.; i Department of Medicine, Vanderbilt University, Nashville, USA.

## Abstract

**Objective:**

To determine the prevalence of adequate monitoring and the costs of measuring CD4+ T-lymphocytes (CD4+ cell) and human immunodeficiency virus (HIV) viral load in people receiving antiretroviral therapy (ART) in seven countries in the WHO Region of the Americas.

**Methods:**

We obtained retrospective, longitudinal data for 14 476 adults who started a first ART regimen at seven HIV clinics in Argentina, Brazil, Chile, Haiti, Honduras, Mexico and Peru between 2000 and 2011. We estimated the proportion of 180-day periods with adequate monitoring, which we defined as at least one CD4+ cell count and one viral load measurement. Factors associated with adequate monitoring were analysed using regression methods. The costs of the tests were estimated.

**Findings:**

The median follow-up time was 50.4 months; the proportion of 180-day periods with adequate CD4+ cell counts was 69% while the proportion with adequate monitoring was 62%. Adequate monitoring was more likely in participants who were older, who started ART more recently, whose first regimen included a non-nucleoside reverse transcriptase inhibitor or who had a CD4+ cell count less than 200 cells/µl at ART initiation. The cost of one CD4+ cell count ranged from 7.37 United States dollars (US$) in Argentina to US$ 64.09 in Chile; the cost of one viral load measurement ranged from US$ 20.34 in Brazil to US$ 186.28 in Haiti.

**Conclusion:**

In HIV-infected participants receiving ART in the WHO Region of the Americas, CD4+ cell count and viral load monitoring was often carried out less frequently than regional guidelines recommend. The laboratory costs of monitoring varied greatly.

## Résumé

**Objectif:**

Vérifier l'existence d'un suivi adéquat et déterminer les coûts liés à la mesure des lymphocytes T CD4+ (cellule CD4+) et de la charge virale en virus de l'immunodéficience humaine (VIH) chez des individus recevant un traitement antirétroviral (TAR) dans sept pays de la Région OMS des Amériques.

**Méthodes:**

Nous avons recueilli des données rétrospectives et longitudinales sur 14 476 adultes ayant commencé un schéma de TAR de première intention dans sept centres de traitement du VIH situés en Argentine, au Brésil, au Chili, à Haïti, au Honduras, au Mexique et au Pérou entre 2000 et 2011. Nous avons estimé la proportion de périodes de 180 jours caractérisées par un suivi adéquat, lequel consistant au minimum en une numération des CD4+ et en une mesure de la charge virale. Les facteurs associés au suivi adéquat ont été analysés à l'aide de méthodes de régression. Une estimation du coût des tests a été effectuée.

**Résultats:**

La durée moyenne du suivi était de 50,4 mois; la proportion des périodes de 180 jours caractérisées par une numération adéquate des CD4+ était de 69% alors que la proportion des périodes caractérisées par un suivi adéquat était de 62%. Le suivi adéquat concernait davantage les participants plus âgés, ayant commencé un TAR plus récemment et dont le schéma de première intention incluait un inhibiteur non nucléosidique de la transcriptase inverse ou dont la numération des CD4+ était inférieure à 200 cellules/µl au début du TAR. Le coût d'une numération des CD4+ variait de 7,37 dollars des États-Unis (US$) en Argentine à 64,09 US$ au Chili; le coût de la mesure d'une charge virale variait de 20,34 US$ au Brésil à 186,28 US$ à Haïti.

**Conclusion:**

En règle générale, le suivi de la numération des CD4+ et de la charge virale chez les participants infectés par le VIH et recevant un TAR dans la Région OMS des Amériques n'était pas réalisé aussi souvent que ce que recommandent les directives régionales. Les frais de laboratoire liés au suivi variaient considérablement.

## Resumen

**Objetivo:**

Determinar la prevalencia de un monitoreo adecuado y los costes de medición de los linfocitos T CD4+ (célula CD4+) y la carga viral del virus de inmunodeficiencia humana (VIH) en personas que reciben terapia antirretroviral (TAR) en siete países de la Región de las Américas de la OMS.

**Métodos:**

Se obtuvieron datos retrospectivos y longitudinales de 14.476 adultos que comenzaban un primer régimen de TAR en siete clínicas de VIH en Argentina, Brasil, Chile, Haití, Honduras, México y Perú entre 2000 y 2011. Se estimó la proporción de periodos de 180 días con un monitoreo adecuado, lo que se definió como al menos un recuento de células CD4+ y una medición de la carga viral. Se analizaron factores relacionados con un monitoreo adecuado mediante la utilización de métodos de regresión. Se estimaron los costes de las pruebas.

**Resultados:**

La mediana de tiempo de monitoreo fue de 50,4 meses; la proporción de periodos de 180 días con recuentos de células CD4+ adecuados fue del 69%, mientras que la proporción de un monitoreo adecuado fue del 62%. Un monitoreo adecuado era más propenso en participantes mayores, que habían comenzado el TAR más recientemente, cuyo primer régimen incluía un inhibidor de la transcriptasa inversa no nucleósido o que tenían un recuento de células CD4+ menor de 200 células/µl en el inicio del TAR. El coste de un recuento de células CD4+ comprendía desde los 7,37 dólares de los Estados Unidos (USD) en Argentina a los 64,09 USD en Chile; el coste de una medición de la carga viral comprendía desde los 20,34 USD en Brasil a los 186,28 USD en Haití.

**Conclusión:**

Entre los participantes infectados por el VIH que recibían el TAR en la Región, el recuento de células CD4+ y la medición de la carga viral eran a menudo realizadas con menor frecuencia que la recomendada por las directrices regionales. Los costes de laboratorio del monitoreo variaban considerablemente.

## ملخص

**الغرض:**

تحديد مدى انتشار المراقبة المناسبة وتكاليف قياس مدى وجود الخلايا اللمفية التائية من فئة CD4+‎ (خلايا فئة CD4+‎) وقياس الحمل الفيروسي المتعلق بفيروس عوز المناعة البشري (HIV) في الأشخاص الذين يتلقون علاجًا باستخدام مضادات الفيروسات القهقرية (ART) في سبع بلدان يشملهم نطاق عمل منظمة الصحة العالمية في الأمريكتين.

**الطريقة:**

حصلنا على بيانات طولانية بأثر رجعي بشأن 14476 من البالغين الذين بدأوا في خطة علاج ART للمرة الأولى في سبع عيادات لعلاج عوز المناعة البشري في الأرجنتين، والبرازيل، وشيلي، وهايتي، وهندوراس، والمكسيك، وبيرو في الفترة بين عام 2000 و2011. وأجرينا تقديرًا لنسبة الفترات التي تبلغ كل منها 180 يومًا والتي توفرت بها طريقة مراقبة مناسبة، والتي عرّفناها بأنها تعداد واحد على الأقل للخلايا فئة CD4+‎‎ وقياسًا واحدًا للحِمل الفيروسي. وتم تحليل العوامل المرتبطة بالمراقبة المناسبة باستخدام طرق التحوف. كما تم تقدير تكاليف الاختبارات.

**النتائج:**

بلغ متوسط فترة المتابعة 50.4 **  **شهرًا؛ ووصلت نسبة الفترات التي تبلغ 180 **  **يومًا والتي تميزت بوجود عدد مناسب من تعداد الخلايا فئة CD4+‎ إلى 69%، بينما بلغت نسبة المراقبة المناسبة 62%. وكان احتمال تطبيق المراقبة المناسبة واردًا بدرجة أكبر في حالة المشاركين الأكبر سنًا ممن بدأوا تلقي ART مؤخرًا، وقد تضمنت خطة العلاج الأولى لهم استخدام أحد الأدوية غير النيوكليوزيدية المضادة لإنزيم المنتسخة المعاكس أو في حالة المشاركين الذين بلغ تعداد الخلايا فئة CD4+‎ لديهم أقل من 200 **  **خلية/ميكرو لتر عند بدء ART. وتراوحت تكلفت إجراء تعداد واحد للخلايا فئة CD4+ من 7.37 **  **دولارًا أمريكيًا في الأرجنتين إلى 64.09 **  **دولارًا أمريكيًا في شيلي؛ وتراوحت تكلفة المرة الواحدة لقياس الحمل الفيروسي من 20.34 **  **دولارًا أمريكيًا في البرازيل إلى 186.28 **  **دولارًا أمريكيًا في هايتي.

**الاستنتاج:**

تم إجراء تعداد الخلايا فئة CD4+‎ ومراقبة الحمل الفيروسي للمشاركين المصابين بعوز المناعة البشري ممن يتلقون ART في المنطقة المشار إليها بشكل أقل تكرارًا في أغلب الأحيان مما ورد في توصيات المبادئ التوجيهية الإقليمية. وقد اختلفت تكاليف المراقبة المختبرية إلى حد كبير.

## 摘要

**目的:**

旨在确定充分监测的普遍性以及测量七个世界卫生组织美洲区域国家中接受抗逆转录病毒治疗 (ART) 者的 CD4+ T 淋巴细胞（CD4+ 细胞）和人体免疫缺损病毒 (HIV) 病毒载量所需的成本。

**方法:**

2000 至 2011 年间 14 476 名成人在阿根廷、巴西、秘鲁、海地、洪都拉斯、墨西哥和智利的七家 HIV 诊所开始了第一个 ART 疗程，我们取得了这些人的回溯、纵向数据。我们估测了为期 180 天的周期在充分监测中所占的比例，我们视之为至少一个 CD4+ 细胞计数和一个病毒载量。 我们利用回归法分析了与充分监测相关的因素， 同时估测了测试的成本。

**结果:**

中位随访期为 50.4 个月，为期 180 天的周期在准确 CD4+ 细胞计数中所占的比例为 69%，而在充分监测中所占的比例为 62%。 更有可能针对老年，最近开始 ART，第一次疗程使用非核苷逆转录酶抑制剂或开始 ART 时 CD4+ 细胞计数少于 200 细胞/µl 的参与人员实施充分监测。 一个 CD4+ 细胞计数的成本从阿根廷 7.37 美元 (US$) 到智利 64.09 美元 (US$) 不等；一个病毒载量的成本从巴西 US$ 20.34 美元 (US$) 到海地 186.28 美元 (US$) 不等。

**结论:**

针对该地区接受 ART 治疗的 HIV 感染者参与人员，展开 CD4+ 细胞计数和病毒载量监测的次数通常少于地区指导方针所建议的次数。 监测的实验室成本差异很大。

## Резюме

**Цель:**

Оценить наличие надлежащего наблюдения, а также стоимость анализа количества T-лимфоцитов CD4+ (клеток CD4+) и концентрации вируса иммунодефицита человека (ВИЧ) в крови у пациентов, получающих антиретровирусную терапию (АРВТ) в семи странах американского региона ВОЗ.

**Методы:**

Были собраны данные ретроспективного долгосрочного исследования 14 476 взрослых пациентов, впервые начавших получать АРВТ в семи клиниках по лечению ВИЧ в Аргентине, Бразилии, Гаити, Гондурасе, Мексике, Перу и Чили в период с 2000 по 2011 г. Оценивалась доля 180-дневных периодов с надлежащим наблюдением, подразумевающим как минимум один анализ на количество клеток CD4+ и одно измерение концентрации вируса. Факторы, связанные с надлежащим наблюдением за пациентами, анализировались с использованием регрессионных методов. Была проведена оценка стоимости анализов.

**Результаты:**

Среднее время последующего врачебного наблюдения составляло 50,4 месяца. Доля 180-дневных периодов адекватного мониторинга числа лимфоцитов CD4+ составила 69%, в то время как доля надлежащего наблюдения составила 62%. Наибольшая вероятность надлежащего наблюдения была выявлена среди участников старшего возраста. Эти пациенты начали АРВТ недавно, либо их первый курс лечения включал прием ненуклеозидных ингибиторов обратной транскриптазы, либо число клеток CD4+ у этих пациентов составляло менее 200 клеток/мкл в начале АРВТ. Стоимость одного анализа для определения числа клеток CD4+ варьировалась от 7,37 долл. США в Аргентине до 64,09 долл. США в Чили. Стоимость одного измерения содержания вируса в крови варьировалась от 20,34 долл. США в Бразилии до 186,28 долл. США на Гаити.

**Вывод:**

У ВИЧ-инфицированных участников исследования, получающих АРВТ в исследуемом регионе, мониторинг числа клеток CD4+ и содержания вируса в крови осуществлялся реже, чем предписывают рекомендации по данному региону. Стоимость расходов на лабораторные исследования значительно различалась между странами.

## Introduction

For people living with human immunodeficiency virus (HIV) who are receiving combination antiretroviral therapy (ART), the most important predictors of treatment outcomes are CD4+ T-lymphocyte (CD4+ cell) count and HIV load in the blood.^1^ Both international and national health organizations recommend that the CD4+ cell count, viral load or both be measured routinely every three to 12 months during ART.^2^–^4^ In Latin America, national clinical guidelines recommend measuring the CD4+ cell count and the viral load every two to six months in people starting ART and then every three to six months once viral suppression has been achieved.^5^–^10^ Recently, the World Health Organization (WHO) updated its guidelines to promote earlier treatment initiation and enhanced monitoring, preferably by viral load testing.^2^

The number of people receiving ART and the cost of treatment are expected to increase in the future because of the worldwide trend to initiate ART at higher CD4+ cell counts and earlier identification of HIV-infected individuals. Improvements in life expectancy resulting from expanded ART programmes are also thought to increase costs.^11^ WHO has been working with regional United Nations agencies and in-country staff and with national ministries of health to support the development and implementation of ART guidelines in individual countries.^12^ At present, however, there is little information on the application of, and adherence to, current WHO and national clinical guidelines in the WHO Region of the Americas. The aims of this study were to determine how frequently the CD4+ cell count and HIV viral load were monitored in people receiving ART in the region between 2000 and 2011. We also wanted to assess the level of adherence to local clinical guidelines and to identify factors associated with infrequent CD4+ cell count and viral load monitoring. We also estimated the cost of CD4+ cell count and viral load measurements in the region.

## Methods

We used retrospective, longitudinal data routinely collected during clinical care and held by the Caribbean, Central and South America Network for HIV Epidemiology (CCASAnet),^13^ which comprises a consortium of adult HIV clinics from seven countries (Argentina, Brazil, Chile, Haiti, Honduras, Mexico and Peru). CCASAnet was established in 2006;^14^ the consortium sites that contributed data to this study are listed in Box 1. All participants who were at least 18 years of age and who initiated their first ART regimen between 1 January 2000 and 31 December 2011 were eligible for inclusion. We excluded participants enrolled in clinical trials to avoid potential bias due to special monitoring practices in these trials.

Box 1 **Participating adult HIV clinic sites from the Caribbean, Central and South America Network for HIV Epidemiology**

Fundación Huésped, Buenos Aires, Argentina.

Instituto de Pesquisa Clinica Evandro Chagas, Fundacão Oswaldo Cruz, Rio de Janeiro, Brazil.

Fundación Arriarán, Santiago, Chile.

Le Groupe Haitien d'Etude du Sarcome de Kaposi et des Infections Opportunistes, Port-au-Prince, Haiti.

Instituto Hondureño de Seguridad Social and Hospital Escuela, Tegucigalpa, Honduras.

Instituto Nacional de Ciencias Médicas y Nutrición Salvador Zubirán, Mexico City, Mexico.

Instituto de Medicina Tropical Alexander von Humboldt, Lima, Peru.

The follow-up period after ART initiation was divided into 180-day periods; follow-up ended on the day of the last recorded visit or when death occurred. Participants were defined as being lost to follow-up if they had not visited the clinic in the 12 months before the database closing date of 1 January 2012. For the primary analysis, treatment monitoring was defined as adequate if at least one CD4+ cell count and one viral load measurement had been made in each 180-day follow-up period. The site in Haiti did not measure viral load during the study period and was not included in the primary analysis. However, the Haitian site was included in a secondary analysis, in which we defined adequate monitoring using the CD4+ cell count alone (in which case, at least one CD4+ cell count had to be measured during each 180-day period).

The primary study outcome was the proportion of 180-day periods during which there was adequate monitoring. Any remaining follow-up time that did not fit within a 180-day period was ignored. For example, if a participant was followed for 400 days, two 180-day periods were included in calculating the proportion of follow-up with adequate monitoring; the remaining 40 days were not included. However, the remaining follow-up periods were included in sensitivity analyses as additional periods. The CD4+ cell count at ART initiation was defined as the measurement closest to the start of ART, but no more than 180 days before or 7 days after the start, and was categorized as either less than 200, 200–350 or more than 350 cells/µL. A participant was described as having had an AIDS-defining event before ART initiation if they had clinical disease that could be classified as Centers for Disease Control and Prevention category C or WHO stage IV. Combination antiretroviral therapy was defined as: (i) therapy based on a non-nucleoside reverse transcriptase inhibitor (e.g. one non-nucleoside reverse transcriptase inhibitor plus two nucleoside reverse transcriptase inhibitors); (ii) protease inhibitor-based therapy, including treatment with one ritonavir-boosted or unboosted protease inhibitor plus two nucleoside reverse transcriptase inhibitors; (iii) triple nucleoside reverse transcriptase inhibitor regimens; or (iv) any other regimen containing at least three drugs. Data were collected at each study site and entered into the database at the local level using codes that did not identify individual participants. Thereafter, data were sent for harmonization to the CCASAnet data coordinating centre at Vanderbilt University, Nashville, United States of America. The coordinating centre also carried out data quality checks and on-site audits to ensure data accuracy. Ethical approval was obtained from institutional review boards at each study site and at Vanderbilt University.

### Statistical analysis

Participant-level factors associated with the frequency of monitoring were identified using Poisson regression: the number of 180-day periods with adequate monitoring was the outcome and the total number of 180-day periods was the offset. A quasi-Poisson estimation procedure was used. Multivariable models included age, sex, CD4+ cell count at ART initiation, year of ART initiation, prior AIDS-defining events and the first ART regimen. Missing data on the CD4+ cell count and prior AIDS-defining events were imputed using multiple imputation with 10 replications. The rate ratio for adequate monitoring was computed for each site and the combined rate ratio across all sites was estimated using random effects meta-analysis. The costs of measuring the CD4+ cell count and viral load were obtained for each site in 2014 in the local currency. Costs were subsequently converted to United States dollars (US$) using exchange rates from the *Wall Street Journal* for Argentina, Brazil, Chile, Mexico and Peru^15^ and rates from local central banks for Haiti^16^ and Honduras.^17^ The median cost of all CD4+ cell counts and viral load measurements per participant per year were estimated for each site. All analyses were performed using R statistical software (R-foundation, Vienna, Austria). Analysis scripts are available from the corresponding author.

## Results

A total of 14 476 participants at the study sites met inclusion criteria: 1285 from Argentina, 2446 from Brazil, 1080 from Chile, 5696 from Haiti, 789 from Honduras, 772 from Mexico and 2408 from Peru. The characteristics of the sites, their sources of funding and the approximate cost of laboratory measurements at each site are summarized in Table 1. The cost of a CD4+ cell count measurement at the different sites ranged from US$ 7.37 in Argentina to US$ 64.09 in Chile and the cost of a viral load measurement ranged from US$ 20.34 in Brazil to US$ 186.28 in Haiti. Guidelines used by all sites recommended measuring the CD4+ cell count every six months or more frequently. Table 2 lists the participants’ demographic characteristics at ART initiation at each site. The median follow-up time was 50.4 months (interquartile range, IQR: 27–82). Overall loss to follow-up was 709 participants (4.9%) ranging from 5% or less in Brazil and Peru to 24.7% in Argentina. The Haitian site provided data only for participants who were not lost to follow-up. There were 967 deaths (6.7%).

**Table 1:** Antiretroviral treatment programmes in seven countries in the WHO Region of the Americas, 2000–2011

Characteristic	Site of adult HIV clinic^a^						
	Argentina	Brazil	Chile	Haiti	Honduras	Mexico	Peru
No. of participants in study	1285	2446	1080	5696	789	772	2408
Start of universal access to ART, year	2000	1991^b^	2003	2003	2003	2002	2004
Type of clinic	Private	Public	Public	NGO	Public	Public	Public
Guidelines used for monitoring ART efficacy	SADI, MOH	MOH	MOH	MOH, PAHO	MOH	MOH	MOH
Recommended periodicity of CD4+ cell count monitoring	3–4 months	3–6 months	3–4 months^c^	6 months	6 months	4–6 months	6 months
Cost of one CD4+ cell count, US$^d^	7.37	17.62	64.09	32.6	14.31 33.39	59.67	38.12^e^
Source of funding for CD4+ cell count monitoring	Refund from Argentine government, social insurance	Brazilian government	Chilean government	PEPFAR, GFATM	Honduran government, social insurance	Mexican government	Peruvian government
Cost of one HIV viral load measurement, US$^d^	55.26	20.34	119.14	186.28^f^	33.39^g^ 160.19^h^	119.27	86.32
Source of funding for HIV viral load monitoring	Refund from Argentine government, social insurance	Brazilian government	Chilean government	Research-funded	Honduran government, social insurance	Mexican government	Peruvian government

ART: antiretroviral therapy; CD4+ cell: CD4+ T lymphocyte; GFATM: Global Fund to Fight AIDS, Tuberculosis and Malaria; HIV: human immunodeficiency virus; MOH: ministry of health; NGO: nongovernmental organization; PAHO: Pan American Health Organization; PEPFAR: The United States President’s Emergency Plan for AIDS Relief; SADI: Sociedad Argentina de Infectología; US$: United States dollar; WHO: World Health Organization.

^a^ Sites participating in the study are listed in Box 1.

^b^ 1991 for monotherapy, 1994 for dual therapy and 1996 for active antiretroviral therapy.

^c^ Every six months for participants with an undetectable viral load, according to the most recent Chilean guidelines (December 2013).

^d^ Costs were converted into United States dollars using exchange rates for 9 July 2014. Exchange rates were obtained from the *Wall Street Journal* for Argentina, Brazil, Chile, Mexico and Peru^15^ and from local central banks for Haiti^16^ and Honduras.^17^

^e^ Cost quoted by the Peruvian National Institute of Health to public institutions.

^f^ Cost for research studies.

^g^ Honduran government cost.

^h^ Honduran social insurance cost.

**Table 2:** Characteristics of participants receiving ART in seven countries in the WHO Region of the Americas, 2000–2011

Participants’ characteristic	Site of adult HIV clinic^a^							
	Argentina (*n* = 1285)	Brazil (*n* = 2446)	Chile (*n* = 1080)	Haiti (*n* = 5696)	Honduras (*n* = 789)	Mexico (*n* = 772)	Peru (*n* = 2408)	Total (*n* = 14 476)
**Age in years, median (IQR)**	39 (33–46)	38 (31–46)	38 (32–45)	39 (32–46)	36 (30–43)	34 (29–42)	35 (29–43)	37 (31–45)
**Male sex, no. (%)**	925 (72)	1611 (66)	952 (88)	2480 (44)	422 (53)	673 (87)	1691 (70)	8754 (60)
**Probable cause of infection, no. (%)**								
Heterosexual sex	340 (26)	1136 (46)	285 (26)	0 (0)	471 (60)	219 (28)	1562 (65)	4013 (28)
Homosexual sex	181 (14)	833 (34)	785 (73)	0 (0)	49 (6)	514 (67)	831 (35)	3193 (22)
Other	55 (4)	81 (3)	9 (1)	0 (0)	3 (0)	17 (2)	13 (1)	178 (1)
Unknown	709 (55)	396 (16)	1 (0)	5696 (100)	266 (34)	22 (3)	2 (0)	7092 (49)
**CD4+ cell count at ART initiation, no. (%)**								
Data missing	306 (24)	426 (17)	302 (28)	772 (14)	151 (19)	132 (17)	326 (14)	2415 (17)
200–350 cells/µL < 200 cells/µL	306 (24)	771 (32)	373 (35)	3035 (53)	447 (57)	355 (45)	1222 (51)	6509 (45)
200–350 cells/µL	349 (27)	650 (27)	234 (22)	1537 (27)	146 (19)	184 (24)	526 (22)	3626 (25)
350 cells/µL	324 (25)	599 (24)	171 (16)	352 (6)	45 (6)	101 (13)	334 (14)	1926 (13)
**Prior AIDS-defining event^b^ at ART initiation, no. (%)**	54 (4)	172 (7)	292 (27)	1223 (21)	252 (32)	332 (43)	848 (35)	3173 (22)
**Prior AIDS-defining event or CD4+ cell count < 200 cells/µL at ART initiation**	335 (26)	871 (36)	541 (50)	3423 (60)	536 (68)	486 (63)	1515 (63)	7707 (53)
**NNRTI-based ART regimen, no. (%)**	869 (68)	1270 (52)	858 (79)	5279 (93)	745 (94)	607 (79)	2024 (84)	11 652 (80)

AIDS: acquired immune deficiency syndrome; ART: antiretroviral therapy; CD4+ cell: CD4+ T lymphocyte; HIV: human immunodeficiency virus; IQR: interquartile range; NNRTI: non-nucleoside reverse transcriptase inhibitor; WHO: World Health Organization.

^a^ Sites participating in the study are listed in Box 1.

^b^ A participant had an AIDS-defining event if they had clinical disease that could be classified as Centers for Disease Control and Prevention category C or WHO stage IV.

### Frequency and cost

The frequency and cost of CD4+ cell counts and viral load measurements varied according to the study site (Table 3). The median frequency of CD4+ cell counts ranged from 2.6 per year in Argentina and Mexico to 1.0 per year in Haiti and the median frequency of viral load measurements ranged from 2.6 per year in Argentina and Mexico to 0.9 per year in Honduras. The annual cost per participant of CD4+ cell count and viral load monitoring was US$ 38 and US$ 140, respectively (Table 3). However the cost varied greatly between sites: the median cost of monitoring the CD4+ cell count ranged from US$ 18 per participant per year in Honduras to US$ 154 in Mexico and the median cost of monitoring the viral load ranged from US$ 41 per participant per year in Brazil to US$ 310 in Mexico. There was no significant correlation between the frequency and cost of CD4+ cell counts (rank correlation: −0.18, *P* = 0.71) or the frequency and cost of viral load measurements (rank correlation: −0.29, *P* = 0.56).

**Table 3:** Antiretroviral treatment monitoring in seven countries in the WHO Region of the Americas, 2000–2011

Monitoring test	Site of adult HIV clinic^a^							
	Argentina (*n* = 1285)	Brazil (*n* = 2446)	Chile (*n* = 1080)	Haiti (*n* = 5696)	Honduras (*n* = 789)	Mexico (*n* = 772)	Peru (*n* = 2408)	Total (*n* = 14 476)
**CD4+ cell count**								
No. of measurements per year, median (IQR)	2.6 (1.9–3.2)	2.1 (1.5–2.6)	1.7 (1.4–2.0)	1.0 (0.7–1.3)	1.3 (0.9–1.6)	2.6 (2.2–3.1)	1.8 (1.4–2.1)	1.5 (0.9–2.1)
Cost per year in US$,^b^ median (IQR)	19 (14–24)	36 (26–45)	112 (87–130)	33 (24–44)	18 (13–22)	154 (132–183)	68 (55–78)	38 (24–63)
**HIV viral load^c^**								
No. of measurements per year, median (IQR)	2.6 (1.9–3.3)	2.0 (1.4–2.5)	1.8 (1.4–2.0)	NA	0.9 (0.6–1.3)	2.6 (2.2–3.1)	1.9 (1.5–2.1)	1.9 (1.4–2.5)
Cost per year in US$,^b^ median (IQR)	145 (106−180)	41 (29–51)	210 (169–240)	NA	138 (91–207)	310 (265–369)	162 (133–184)	140 (52–194)

CD4+ cell: CD4+ T lymphocyte; HIV: human immunodeficiency virus; IQR: interquartile range; NA: not applicable; US$: United States dollar; WHO: World Health Organization.

^a^ Sites participating in the study are listed in Box 1.

^b^ Costs were converted into United States dollars using exchange rates for 9 July 2014. Exchange rates were obtained from the *Wall Street Journal* for Argentina, Brazil, Chile, Mexico and Peru^15^ and from local central banks for Haiti^16^ and Honduras.^17^

^c^ Viral load was assessed by measuring the plasma HIV-RNA level.

### Adherence to guidelines

**Fig. 1:**
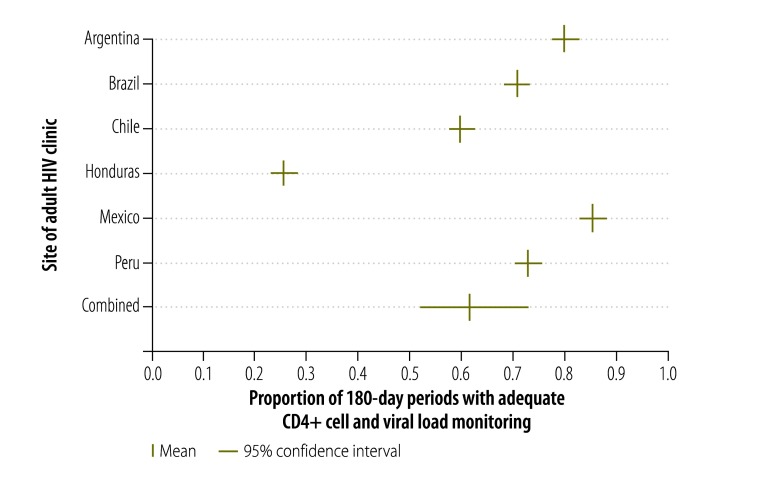
Adequate CD4+ cell count and HIV viral load monitoring in six countries in the WHO Region of the Americas, 2000–2011

**Fig. 2:**
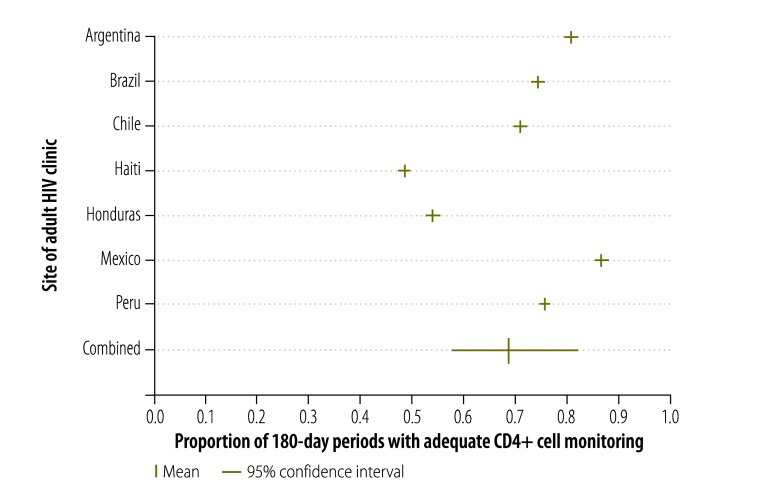
Adequate CD4+ cell count monitoring in seven countries in the WHO Region of the Americas, 2000–2011

The adequacy of CD4+ cell count and viral load monitoring is shown for each site in Fig. 1. The proportion of periods with adequate monitoring was highest in Mexico (86%) and Argentina (80%) but much lower in Honduras (26%). Across the six sites for which data were available, the proportion of periods with adequate CD4+ cell count and viral load monitoring was 62% (95% confidence interval, CI: 52–73) – the large confidence interval was due to the substantial variation between sites. Fig. 2 shows the proportion of 180-day periods with adequate CD4+ cell count monitoring alone. Overall, the proportions were higher than those observed for CD4+ cell count and viral load monitoring combined and ranged from 86% and 81% in Mexico and Argentina, respectively, to 54% and 48% in Honduras and Haiti, respectively. Across all seven sites, the proportion of periods with adequate CD4+ cell count monitoring alone was 69% (95% CI: 57–82). General trends were similar in sensitivity analyses that included periods of less than 180 days (data available from corresponding author).

CD4+ cell: CD4+ T lymphocyte; HIV: human immunodeficiency virus; WHO: World Health Organization.

Notes: Monitoring was defined as adequate if at least one CD4+ cell count and one viral load measurement were made in each 180-day follow-up period. Sites participating in the study are listed in Box 1. The site in Haiti did not assess viral load during the study period.

CD4+ cell: CD4+ T lymphocyte; HIV: human immunodeficiency virus; WHO: World Health Organization.

Notes: Monitoring was defined as adequate if at least one CD4+ cell count measurement was made in each 180-day follow-up period. Sites participating in the study are listed in Box 1.

**Fig. 3:**
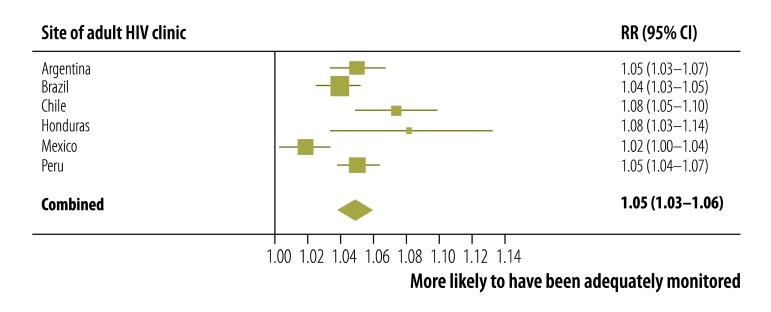
Adequate CD4+ cell count and HIV viral load monitoring in six countries in the WHO Region of the Americas, 2000–2011: rate ratio for a 10-year increase in age

**Fig. 4:**
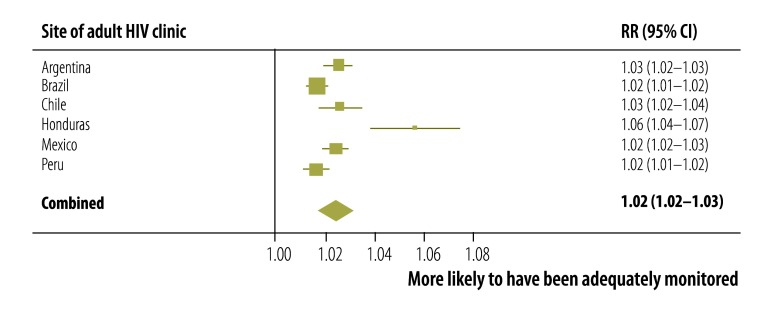
Adequate CD4+ cell count and HIV viral load monitoring in six countries in the WHO Region of the Americas, 2000–2011: rate ratio for a 1-year reduction in time since ART initiation

ART: antiretroviral therapy; CD4+ cell: CD4+ T lymphocyte; CI: confidence interval; HIV: human immunodeficiency virus; RR: rate ratio; WHO: World Health Organization.

Notes: Monitoring was defined as adequate if at least one CD4+ cell count and one viral load measurement were made in each 180-day follow-up period. Points to the right of the vertical line indicate that participants who started ART more recently are more likely to be adequately monitored. RR per calendar year increase in the date of ART initiation. Sites participating in the study are listed in Box 1. The site in Haiti did not assess viral load during the study period.

**Fig. 5:**
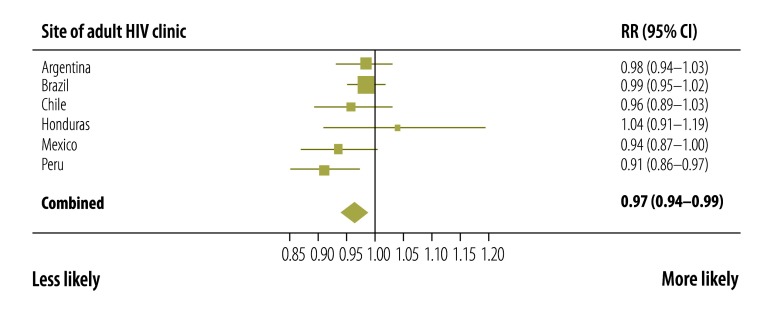
Adequate CD4+ cell count and HIV viral load monitoring in six countries in the WHO Region of the Americas, 2000–2011:rate ratio for CD4+ T-cell count at ART initiation >350 cells/ µL versus <200 cells/µL

ART: antiretroviral therapy; CD4+ cell: CD4+ T lymphocyte; CI: confidence interval; HIV: human immunodeficiency virus; RR: rate ratio; WHO: World Health Organization.

Notes: Monitoring was defined as adequate if at least one CD4+ cell count and one viral load measurement were made in each 180-day follow-up period. Points to the left of the vertical line indicate that participants with higher cell count at ART initiation are less likely to be adequately monitored. Sites participating in the study are listed in Box 1. The site in Haiti did not assess viral load during the study period.

**Fig. 6:**
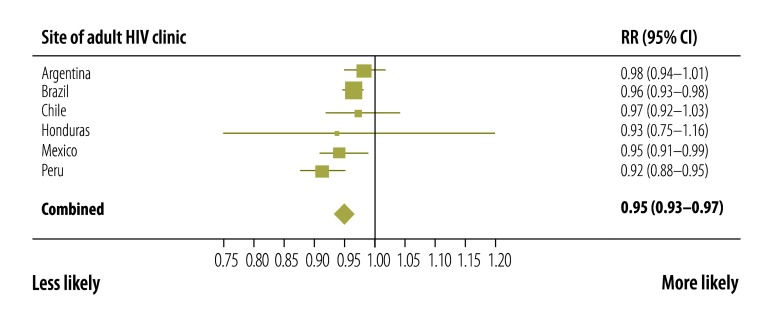
Adequate CD4+ cell count and HIV viral load monitoring in six countries in the WHO Region of the Americas, 2000–2011: rate ratio for nucleoside reverse transcriptase inhibitor regimen versus NNRTI regimen

ART: antiretroviral therapy; CD4+ cell: CD4+ T lymphocyte; CI: confidence interval; HIV: human immunodeficiency virus; NNRTI: non-nucleoside reverse transcriptase inhibitor; RR: rate ratio; WHO: World Health Organization.

Notes: Monitoring was defined as adequate if at least one CD4+ cell count and one viral load measurement were made in each 180-day follow-up period. Points to the left of the vertical line indicate that participants who were on a NNRTI regimen were less likely to be inadequately monitored. Sites participating in the study are listed in Box 1. The site in Haiti did not assess viral load during the study period.

Factors associated with adequate CD4+ cell count and viral load monitoring at each site and across all sites combined were identified. At all sites, adequate CD4+ cell count and viral load monitoring was more likely in older participants (Fig. 3) and in those who started ART more recently (Fig. 4). Patients with a CD4+ cell count less than 200 cells/µL at ART initiation were more likely to have adequate monitoring than those with a count more than 350 cells/µL (Fig. 5). Participants whose first ART regimen contained a non-nucleoside reverse transcriptase inhibitor were also more likely to have adequate monitoring than those treated with other regimens (Fig. 6). Neither sex, a CD4+ cell count in the range 200 to 350 cells/µL nor prior AIDS-defining events influenced the likelihood of adequate CD4+ cell count and viral load monitoring.

CD4+ cell: CD4+ T lymphocyte; CI: confidence interval; HIV: human immunodeficiency virus; RR: rate ratio; WHO: World Health Organization.

Notes: Monitoring was defined as adequate if at least one CD4+ cell count and one viral load measurement were made in each 180-day follow-up period.

Points to the right of the vertical line indicate that older participants are more likely to be adequately monitored. Sites participating in the study are listed in Box 1. The site in Haiti did not assess viral load during the study period.

**Fig. 7:**
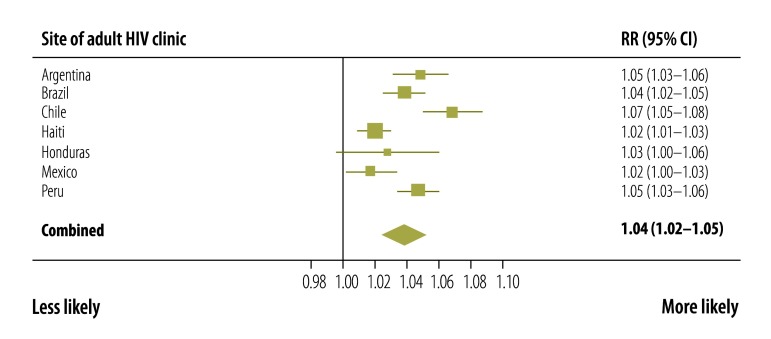
Adequate CD4+ cell count monitoring in seven countries in the WHO Region of the Americas, 2000–2011: rate ratio for a 10-year increase in age

**Fig. 8:**
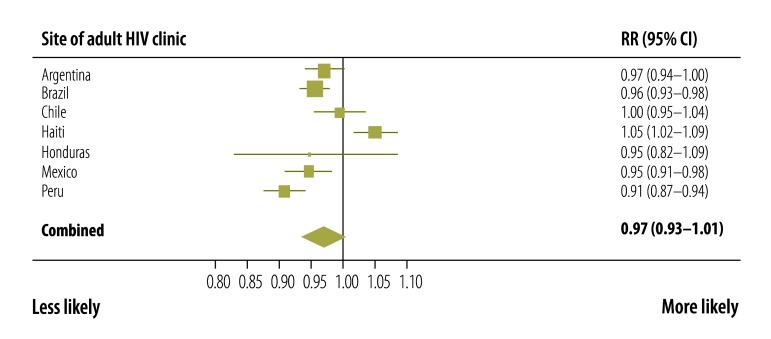
Adequate CD4+ cell count monitoring in seven countries in the WHO Region of the Americas, 2000–2011

ART: antiretroviral therapy; CD4+ cell: CD4+ T lymphocyte; CI: confidence interval; HIV: human immunodeficiency virus; NNRTI: non-nucleoside reverse transcriptase inhibitor; RR: rate ratio; WHO: World Health Organization.

Notes: Monitoring was defined as adequate if at least one CD4+ cell count measurement was made in each 180-day follow-up period. Points to the left of the vertical line indicate that participants who were on a NNRTI regimen were less likely to be inadequately monitored. Sites participating in the study are listed in Box 1.

Factors associated with adequate CD4+ cell count monitoring alone were also identified. Again, adequate monitoring was more likely in older participants (Fig. 7) and in those whose first ART regimen contained a non-nucleoside reverse transcriptase inhibitor (Fig. 8). Sensitivity analyses that included periods shorter than 180 days yielded similar results (details available from the corresponding author). Neither sex, prior AIDS-defining events, nor the year of ART initiation influenced the likelihood of adequate CD4+ cell count monitoring, though there was some indication that a CD4+ cell count less than 200 cells/µL at ART initiation may have had an effect.

CD4+ cell: CD4+ T lymphocyte; CI: confidence interval; HIV: human immunodeficiency virus; RR: rate ratio; WHO: World Health Organization.

Notes: Monitoring was defined as adequate if at least one CD4+ cell count measurement was made in each 180-day follow-up period. Points to the right of the vertical line indicate that older participants are more likely to be adequately monitored. Sites participating in the study are listed in Box 1.

## Discussion

We found that HIV-infected people starting ART at seven sites in the region were monitored less frequently than recommended by regional and national clinical guidelines. On average, CD4+ cell count and viral load monitoring were done at least once every six months for only 62% of the time participants were in care, although the proportion varied greatly across sites. The equivalent proportion for CD4+ cell count monitoring alone was 69%. Adequate monitoring of both parameters was associated with older age at ART initiation, receiving ART in more recent years, starting ART when the CD4+ cell count was less than 200 cells/µL and a first ART regimen that included a non-nucleoside reverse transcriptase inhibitor.

The annual cost of CD4+ cell count and viral load monitoring per participant varied greatly between countries. For instance, the annual cost of CD4+ cell count monitoring per participant in Mexico was around four times the median cost in the region and almost nine times the cost in Honduras. Although we are not aware of previous studies of the cost of monitoring ART efficacy in the region, the Pan American Health Organization (PAHO) recently noted that the annual cost of ART medications per participant in the most expensive countries was approximately 10 times that in the lowest-cost countries.^18^ These differences have been attributed to the varying use of generic drugs and to the ART procurement mechanism preferred by each country.^18^,^19^

The implications of the variability in monitoring costs across the region we observed are difficult to discern. For instance, although it appears that the cost of CD4+ cell count and viral load monitoring at individual sites was not related to the frequency of monitoring or to adherence to guideline recommendations, we were not able to assess how cost differences affected participant care, such as the ability to detect treatment failure. Furthermore, it would be difficult to use our results to identify a single initiative that could reduce costs across the region because each centre itself negotiates costs and budgets with local laboratories and with different sponsors. For the large centres in Argentina, Brazil, Chile and Peru, it may be possible to reduce costs by exploiting the large demand to leverage services. However, this strategy may not work for centres in Haiti, Honduras or Mexico, where government and international funding agencies, or the local infrastructure, may restrict their ability to negotiate costs locally.

The frequency of CD4+ cell count and viral load monitoring we observed in HIV-infected persons receiving ART was very similar to that reported in the region by PAHO.^20^ It is possible to divide our sites into two groups: those where participants were followed up in accordance with clinical guidelines most of the time (i.e. Argentina, Brazil, Chile, Mexico and Peru) and those where participants were followed up in accordance with guidelines less than half the time (i.e. Haiti and Honduras). It is noteworthy that the proportion of periods when CD4+ cell count and viral load monitoring was adequate was very similar to the proportion when CD4+ cell count monitoring alone was adequate at all sites except Haiti, which did not measure viral load, and Honduras, where in practice laboratory monitoring of the efficacy of ART was based more on CD4+ cell counts alone, than on both CD4+ cell count and viral load monitoring.

The low level of adherence to clinical guidelines we observed was probably due to a combination of factors related to the participants, physicians and other health-care providers and, more generally, to structural and programmatic characteristics. At the individual level, our findings agree with those of previous studies in identifying older age as an important factor associated with adherence to ART,^21^ adequate CD4+ cell count monitoring, retention on treatment and fewer missed clinic visits.^22^,^23^ We found that a CD4+ cell count less than 200 cells/µL at ART initiation was associated with adequate CD4+ cell count monitoring overall, but not at most individual sites. We hypothesize that clinicians’ perception that profound immunosuppression is associated with an increased risk of complications may have led to more frequent monitoring.

Although we were not able to evaluate structural and programmatic characteristics in detail, we observed several factors that could explain some of the differences in monitoring between sites. For example, the Global Fund to Fight AIDS, Tuberculosis and Malaria did not cover the cost of viral load measurements in Haiti and, consequently, this test was little used. Haiti and Honduras have generalized HIV epidemics, whereas in the rest of the region HIV infection is concentrated among men who have sex with men and injection-drug users.^24^ There were also clear differences in national income, development indices and health expenditure per capita^25^ between Haiti and Honduras and the other countries we studied.^25^,^26^ Although the maturity of universal ART access programmes could have influenced adherence to clinical guidelines, most countries in the region, including Haiti and Honduras, expanded their programmes between 2002 and 2003 and quickly ensured that most people in need of ART received treatment. Finally, the site in Haiti has been actively involved in emergency responses to the earthquake in 2010 and to the ensuing cholera epidemic in 2012,^27^ which may have diverted resources from HIV-related health care. It is possible that viral load monitoring in Haiti could be increased if the Global Fund updated its policy on funding monitoring or PAHO changed its recommendations on monitoring. However, detailed research on differences in treatment programmes across a larger number of centres is needed to clearly identify policies that could improve adherence to clinical guidelines and reduce costs.

Our study has several limitations. First, we used a conservative definition of adequate CD4+ cell count and viral load monitoring – six months is currently the maximum time interval recommended by the guidelines used at all sites and most countries in the region recommend measurements every three to four months.^5^–^10^ Thus, we may have overestimated adherence to recommendations. Since we were not able to identify reasons for poor adherence or for the wide range in monitoring costs, it is difficult to propose interventions that would improve adherence or decrease costs. Another limitation is that it is not clear whether poor adherence to laboratory monitoring guidelines was associated with worse outcomes in HIV-infected people in the region. Certainly, virological failure will be detected less often if fewer viral load measurements are done and this may lead to poorer clinical outcomes. Earlier research at our study sites found that mortality was generally higher in Haiti and Honduras,^28^ where monitoring was less frequent, but the high mortality was probably due to several factors and not to monitoring practices alone. Previous studies in Africa and mathematical modelling suggest that carrying out laboratory monitoring less frequently than recommended may have negative consequences for the health of people on ART and that measuring the viral load two or three times a year may be more cost-effective and reduce HIV transmission.^29^,^30^ However, the clinical and cost benefits of measuring both the CD4+ cell count and viral load as frequently as currently recommended are still debated.^31^–^35^ A randomized trial may be required to resolve the issue.

In conclusion, adherence to recommended CD4+ cell count and viral load monitoring frequencies was generally poor in the region, with large variations between sites. Laboratory costs were also highly variable. Further research is needed to identify the underlying cause of differences in the frequency and cost of monitoring across the region and studies should be carried out to evaluate the impact of less frequent laboratory monitoring on health outcomes in HIV-infected persons receiving ART. We hope our study findings will be helpful for developing and implementing HIV treatment guidelines.
